# Acceptability of magnetic resonance imaging for prostate cancer diagnosis with patients and GPs: a qualitative interview study

**DOI:** 10.3399/BJGP.2023.0083

**Published:** 2024-04-03

**Authors:** Samuel WD Merriel, Stephanie Archer, Alice S Forster, David Eldred-Evans, John S McGrath, Hashim U Ahmed, Willie Hamilton, Fiona M Walter

**Affiliations:** Centre for Primary Care and Health Services Research, University of Manchester, Manchester; Department of Health and Community Sciences, University of Exeter, Exeter.; Department of Public Health and Primary Care, University of Cambridge, Cambridge; Department of Psychology, University of Cambridge, Cambridge.; Our Future Health, London.; Department of Surgery and Cancer, Imperial College London, London.; Department of Urological Surgery, Royal Devon University Healthcare NHS Foundation Trust, Exeter.; Department of Surgery and Cancer, Imperial College London, London.; Department of Health and Community Sciences, University of Exeter, Exeter.; Wolfson Institute of Population Health, Queen Mary University of London, London.

**Keywords:** diagnosis acceptability, diagnostic techniques and procedures, primary health care, prostate, prostate cancer, magnetic resonance imaging

## Abstract

**Background:**

Magnetic resonance imaging (MRI) of the prostate is a new, more accurate, non-invasive test for prostate cancer diagnosis.

**Aim:**

To understand the acceptability of MRI for patients and GPs for prostate cancer diagnosis.

**Design and setting:**

Qualitative study of men who had undergone a prostate MRI for possible prostate cancer, and GPs who had referred at least one man for possible prostate cancer in the previous 12 months in West London and Devon.

**Method:**

Semi-structured interviews, conducted in person or via telephone, were audio-recorded and transcribed verbatim. Deductive thematic analysis was undertaken using Sekhon’s Theoretical Framework of Acceptability, retrospectively for patients and prospectively for GPs.

**Results:**

Twenty-two men (12 from Devon, age range 47–80 years), two patients’ partners, and 10 GPs (6 female, age range 36–55 years) were interviewed. Prostate MRI was broadly acceptable for most patient participants, and they reported that it was not a significant undertaking to complete the scan. GPs were more varied in their views on prostate MRI, with a broad spectrum of knowledge and understanding of prostate MRI. Some GPs expressed concerns about additional clinical responsibility and local availability of MRI if direct access to prostate MRI in primary care were to be introduced.

**Conclusion:**

Prostate MRI appears to be acceptable to patients. Some differences were found between patients in London and Devon, mainly around burden of testing and opportunity costs. Further exploration of GPs’ knowledge and understanding of prostate MRI could inform future initiatives to widen access to diagnostic testing in primary care.

## Introduction

Prostate cancer is the most common cancer diagnosed in men in the UK.^1^ In recent years, significant challenges have arisen concerning the overdiagnosis of clinically insignificant prostate cancer — which is unlikely to affect a man’s health — and the limited accuracy of available diagnostic tests. Until recent years, the gold standard diagnostic test for prostate cancer has been a transrectal ultrasound-guided (TRUS) biopsy of the prostate. TRUS biopsy procedures take at least 6–12 samples from different regions of the prostate, which are then examined by a histopathologist for signs of prostate cancer.^2^ TRUS biopsy carries a significant risk of infection and sepsis, and there is evidence of understaging and missed diagnoses as a result of the random nature of sampling the prostate using this approach.^3^

Pre-biopsy magnetic resonance imaging (MRI) scanning of the prostate for suspected prostate cancer has recently emerged as a new diagnostic test. Based on evidence showing it is more accurate for clinically significant prostate cancer, and can also be used to avoid unnecessary prostate biopsies in some men,^4^^,^^5^ national and international guidelines have been updated to recommend pre-biopsy MRI be incorporated into prostate cancer diagnostic pathways.^2^^,^^6^ Within the NHS in England, prostate MRI is usually performed in secondary care following an urgent suspected cancer referral by a patient’s GP. Despite recent policy initiatives to widen access to diagnostic tests for cancer from primary care,^7^ prostate MRI is not currently available for direct access by GPs.

**Figure 1.**
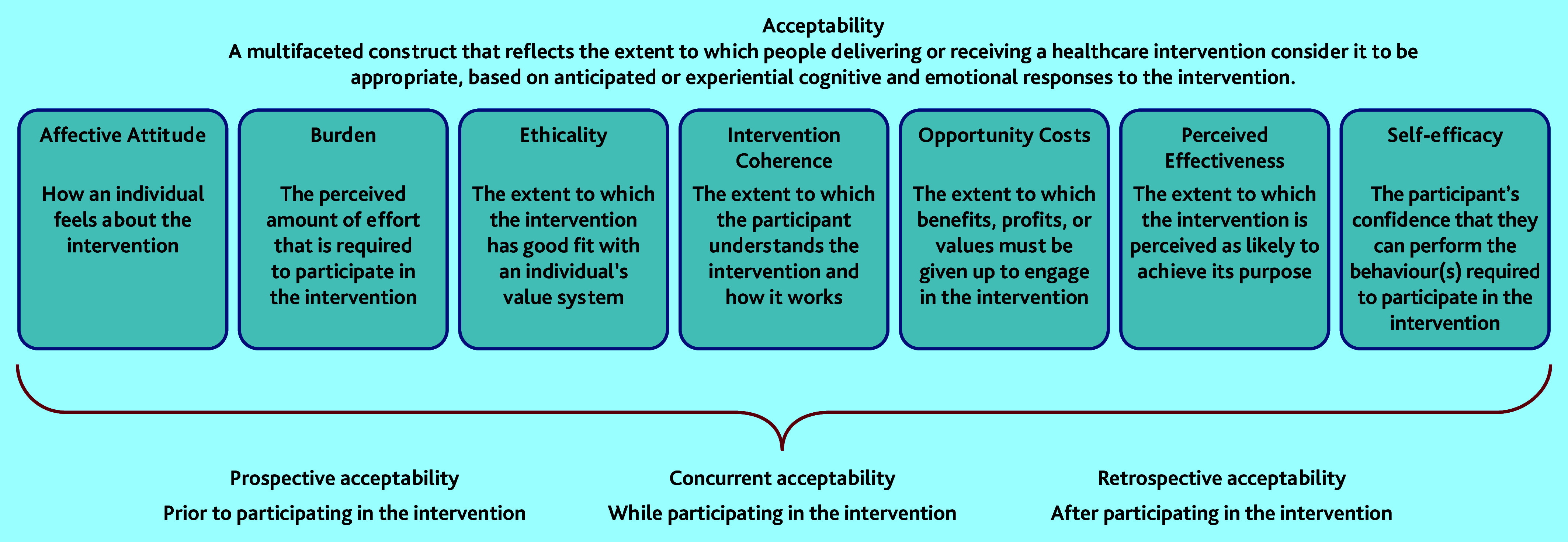
Sekhon’s Theoretical Framework of Acceptability.^15^ © Sekhon *et al.* Adapted under CC BY 4.0 (https://creativecommons.org/licenses/by/4.0/).

Implementation of new diagnostic tests into routine clinical practice should ideally follow a rigorous process of evaluation. Frameworks for assessing and evaluating tests suggest the test should demonstrate more patient benefit than harm; be cost-effective relative to currently available tests; be able to be integrated into the diagnostic pathway; and be acceptable to patients and clinicians.^8^^–^^13^ Acceptability of diagnostic tests has been measured in a number of ways, but no agreed definition for acceptability exists.^14^ Sekhon *et al* recently proposed a Theoretical Framework of Acceptability (TFA) relating to healthcare interventions, which includes seven key constructs (Figure 1): affective attitude, burden, ethicality, intervention coherence, opportunity costs, perceived effectiveness, and self-efficacy.^15^ This framework is intended to be applicable to both patients and clinicians involved in healthcare interventions, and can be applied prospectively, concurrently, or retrospectively.

**Table:** How this fits in

Existing tests for prostate cancer available in primary care have significant limitations. Recent NHS policy initiatives to improve early cancer diagnosis have in part focused on widening access to diagnostic testing, including magnetic resonance imaging (MRI). This qualitative interview study assessed the acceptability of prostate MRI using Sekhon’s Theoretical Framework of Acceptability, with a diverse sample of patients who had undergone prostate MRI and GPs who had recently referred patients with suspected prostate cancer. GPs’ knowledge and understanding of the role of prostate MRI varied widely, with concerns raised about access to MRI and additional workload on primary care.

Two studies have assessed some aspects of patient acceptability of MRI tests for prostate cancer using questionnaires assessing side effects and attitudes.^16^^,^^17^ Few studies exist that explore GPs’ views about prostate cancer tests. To the authors’ knowledge, there are no studies that examine acceptability of any diagnostic test for prostate cancer with a theoretical underpinning, and questions remain about men’s experience of undergoing a prostate MRI and receiving the results. The aim of this study was to understand, from the perspective of patients and GPs, the acceptability of MRI for men as a diagnostic test for prostate cancer in two different diagnostic pathway designs. Sekhon’s definition of acceptability was adopted for this study (Figure 1).

## Method

This qualitative study employed semi-structured interviews with men referred from primary care with possible prostate cancer who had undergone an MRI scan, and GPs who have recently referred men with possible prostate cancer for further investigation. This study formed part of a PhD programme of research assessing the potential impact of prostate MRI on the primary care element of the prostate cancer diagnostic pathway. The interviews explored the acceptability of prostate MRI among patients and GPs (the focus of this study), and their experiences of the current diagnostic pathway as a whole (findings reported elsewhere).^18^

It was anticipated that participant experiences of the prostate cancer diagnostic pathway and the diagnostic tests would be informed by participants’ personal characteristics, the social context, the diagnostic pathway followed, and interaction with others. As such, a constructivist approach underpinned the research design and analysis.^19^

### Participants

This study recruited participants from two populations:

- patients with possible prostate cancer who had undergone an MRI scan as part of their diagnostic workup; and
- GPs who had referred at least one male patient for investigation for possible prostate cancer within the preceding 12 months.

Patients who already had a diagnosis of prostate cancer and were undergoing MRI for active surveillance or watchful waiting were not invited to participate, as the focus of this study was on the role of MRI in the diagnosis of prostate cancer, rather than management. GPs and patients were recruited separately, and not in dyads.

### Recruitment

**Figure 2.**
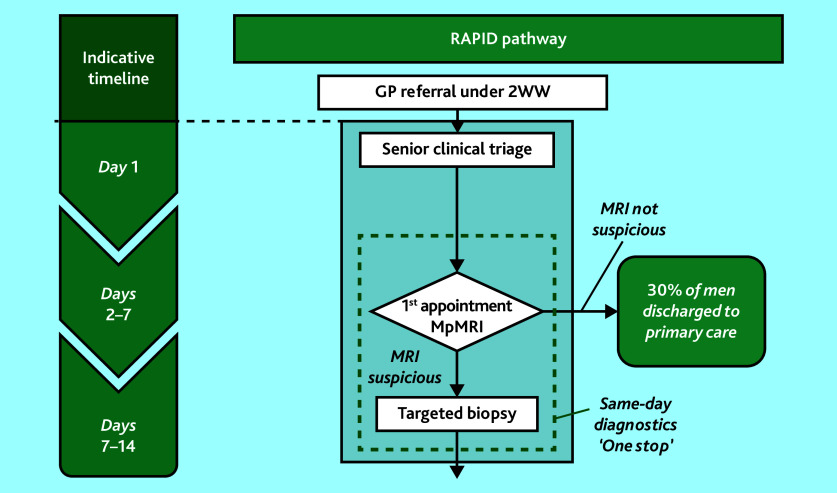
The RAPID pathway, Imperial College Healthcare NHS Trust. 2WW = two week wait. mpMRI = multiparametric MRI. RAPID = Rapid Access to Prostate Imaging and Diagnosis.

A purposive sampling approach was taken for participant recruitment to obtain as diverse a group of participants and experiences as possible. Patients were recruited from two NHS Trusts with contrasting diagnostic pathways: the Imperial College Healthcare NHS Trust in London, and the Royal Devon University Healthcare NHS Foundation Trust in Exeter. Imperial College employs the Rapid Access to Prostate Imaging and Diagnosis (RAPID) pathway (Figure 2), where patients undergo a prostate MRI scan, receive their MRI result, and potentially undergo a prostate biopsy at a single outpatient attendance. The Royal Devon and Exeter Hospital uses separate outpatient appointments for a prostate MRI, consultant review, and prostate biopsy if needed (herein referred to as ‘traditional’). Research staff identified potentially eligible men, and contacted them within days of undergoing an MRI scan to discuss this study and offer them a participant information leaflet (PIL). Patients were offered the option of having another person present for part or all of the interview if they wanted. Reasonable travel costs for patient participants to attend a face-to-face interview were reimbursed, and participants were offered a £20 gift voucher in recognition of contributing their time to participate in the study.

GPs were recruited via two National Institute for Health and Care Research (NIHR) Clinical Research Networks (CRNs) in the same areas as the patients and their respective prostate cancer diagnostic pathways: the North West London CRN and the South West Peninsula CRN. The CRNs identified local practices from which to recruit eligible GPs. Selected GPs were given a PIL to consider participating in the study. GP practices were reimbursed for the GP’s time to participate in an interview.

### Data collection

One-to-one interviews were conducted for all participants between July and November 2019 by one of the authors. Patient participants were mostly interviewed face-to-face in their own home. All GP participant interviews were conducted via telephone. A semi-structured approach was followed, with separate interview topic guides for patient and GP interviews to support discussions (see Supplementary Information S1 and S2). The topic guide was developed to incorporate all aspects of the current prostate cancer diagnostic pathway in the UK, not just MRI in isolation. The topic guide was used flexibly within the interviews, to try to ensure that no key aspects of the diagnostic pathway experience were missed. An encrypted audio-recording device was employed to record all interviews, and written notes were taken during and immediately following the interviews. All interview recordings were professionally transcribed. Interview times ranged from 15–32 minutes for GPs and 16–45 minutes for patients.

### Data analysis

Transcriptions were imported into NVivo (version 12) to manage the data for the analysis. The researchers initially immersed themselves in the data through reading and re-reading individual transcripts and listening back to the audio-recordings of the interviews. For the purposes of this analysis, a selection of early interviews were coded deductively using Sekhon’s TFA, and this initial codebook was reviewed and refined by three authors. The remaining interview transcripts were coded using the codebook developed.^19^ Coded data were grouped under the constructs of Sekhon’s TFA for analysis. Prostate MRI acceptability was assessed retrospectively with patients, and in a prospective, exploratory manner with GPs (exploring how they felt about the potential for direct access to prostate MRI from primary care).

### Patient and public involvement (PPI)

A PPI group of eight men from South West England was recruited via the People in Health West of England (PHWE) initiative; it included men with a range of ages, ethnic backgrounds, and experiences with prostate cancer. PPI group members reviewed the plain English summary and all patient participant documents, and gave feedback before submission as part of the ethical approval application. They also gave input into the interview topic guides and discussed themes emerging from a single anonymised text. A study summary report was sent to all study participants after all data had been collected and analysed.

### COREQ reporting guidelines

This manuscript has been written in accordance with the consolidated criterion for reporting qualitative research (COREQ) checklist.^20^

## Results

### Participants

Twenty-two patients were interviewed; two patient participants’ partners also contributed. Patient participant ages ranged from 47–80 years, and 12 lived in Devon where the traditional pathway was followed. All patients had undergone a prostate MRI within weeks of the interview. Six participants had a diagnosis of prostate cancer at the time of the interview. Ten GPs were interviewed: most were female (*n* = 6) and GP partners (*n* = 8) (Table 1). Three potential patient participants and two GPs declined to participate after being approached.

**Table 1.** Patient and GP demographics

	**Patients (*n* = 22),** ***n***		**GPs (*n* = 10),** ***n***
**Age, years**		**Age, years**	
<65	8	31–40	3
≥65	14	41–50	6
		≥50	1

**Local pathway**		**Local pathway**	
RAPID	10	RAPID	4
Traditional	12	Traditional	6

**Ethnicity**		**Gender**	
White	19	Male	4
BME	3	Female	6

**PIRADS v2**		**Role**	
1–2	6	Partner	8
3–5	15	Salaried	2
Unknown	1		

*BME = Black and minority ethnic background. PIRADS = prostate imaging reporting and data system (1–2 = normal, 3–5 = abnormal). RAPID = Rapid Access to Prostate Imaging and Diagnosis.*

### Acceptability of MRI for possible prostate cancer

Affective attitude refers to how the individual receiving or delivering the intervention felt about it. The vast majority of patient interviewees and their partners were positive about the idea of having an MRI scan of their prostate. They were quite happy to undergo a scan, and would do so again if required:

*‘I’d go for any scan, anything like that. Needles don’t bother me, scans don’t bother me.’*

(Patient [P]21, traditional pathway, aged ≥65 years)

*‘And so I think, if that can show up cancer and prostate and stuff like that, it’s a brilliant piece of kit and I think it should be used as much as it can be.’*

(P03’s partner, traditional pathway, aged <65 years)

A minority of GPs felt similarly positive about the concept, with others expressing no opinion or raising concerns about additional workload:

*‘I think it will be a really useful idea.’*

(GP03, male, RAPID pathway)

GPs also considered the potential of MRI from the patient’s perspective. They identified the non-invasive nature of the test, compared with biopsy procedures, as an attractive attribute for their patients:

*‘I guess, I would welcome something that would be non-invasive for patients because that’s always good.’*

(GP05, female, traditional pathway)

Burden refers to how much effort is involved in participating in the intervention. Most, but not all, patients reported that undergoing an MRI scan of the prostate was not a significant undertaking:

*‘Whereas the scans just takes a bit of time, and it’s no hassle at all.’*

(P05, RAPID pathway, aged ≥65 years)

*‘To be honest with you, when I had that done, it bloody vibrated … and when I had it done at* [hospital name] *it thumped and bumped and, you know … and it is a little bit traumatic, you know?’*

(P18, traditional pathway, aged ≥65 years)

Patient interviewees reported a range of views towards the amount of time required to be inside the MRI scanner, ranging from 20 to 45 minutes. Claustrophobia and being in a small, enclosed space for a period of time was a challenge for some patients:

*‘Mainly because I suffer from claustrophobia. And the first one, because it was lower back, I was pretty much inside the machine, which I did not enjoy*.*’*

(P23, traditional pathway, aged ≥65 years)

From the GP’s perspective, concerns around the burden of MRI focused on the potential for increased clinical responsibility and workload, as well as increased demand for MRI from patients if GPs were able to order the test:

*‘… but if I have to have another conversation with somebody about the pros and cons of whether they want to go see a urologist, have a biopsy, have a PSA* [prostate-specific antigen test]*, or have an MRI scan, that’s not so great really. That’s just another conversation about a complex thing that I’m going to have to try and weigh up for the patient*.*’*

(GP05, female, traditional pathway)

Ethicality explores how the intervention fits with an individual’s value system. From the data gathered there did not seem to be any significant conflicts on a personal level for patients undergoing an MRI scan, or GPs referring patients onto a pathway where they would have an MRI scan.

Intervention coherence covers the participant’s understanding of the intervention and how it works. Levels of understanding of the technical aspects of MRI and the specific role of MRI in the prostate cancer diagnostic pathway varied widely between the patient and GP participants. Patients had a lower level of knowledge generally, whereas GP understanding ranged from very limited to being well informed about why MRI is used for prostate cancer. The underlying reasons for this variation in understanding differ between the two groups. Patients are not medically trained and, therefore, may need to rely on healthcare staff to explain a procedure clearly, whereas GPs’ understanding appeared to depend on their clinical experience and local access to MRI:

*‘The MRI scan basically found some areas that were, let’s say, suspicious. I don’t think they exactly found cancer but … ’*

(P02, traditional pathway, aged ≥65 years)

*‘I don’t know quite how an MRI of the prostate is done, how much you have to … MRI, how long it takes. I don’t know all of that.’*

(GP08, female, RAPID pathway)

*‘I think that the real … that is more relevant or perhaps most relevant if you’re considering using MRI to avoid biopsy. So actually selecting patients out who’ve got the lowest risk* [of] *disease just on the basis of MRI images without biopsy*.*’*

(GP10, male, traditional pathway)

Opportunity costs explores what an individual must give up in order to engage with the intervention. Some patients had to invest a significant amount of time for travel and car parking at hospitals, where most MRI scanners are located:

*‘So you’ve seen where we live and what parking is like in bloody … the hospital, excuse my language, sorry, the hospital, I left here … I had a nine-thirty appointment, yeah … no I had a ten-thirty appointment, was it something like that? No, nine … whatever it was … Anyway, I got there an hour early, at least an hour early, you know, to find a parking space.’*

(P18, traditional pathway, aged ≥65 years)

For GPs, the opportunity costs related to concerns around overloading the system if MRI was made accessible in primary care:

*‘The only thing if MRI became more … if an MRI for prostate became more access … became accessible to GPs I think there probably is a risk that we would be under pressure to be referring people asymptomatically, who are educated people who want to just have an MRI to be sure. And I think that … that’s not a great thing. I would be quite resistant to that.’*

(GP07, female, RAPID pathway)

Perceived effectiveness relates to how the participant perceives the likelihood of the intervention achieving its purpose. Patients generally had confidence in MRI as a test for possible prostate cancer, and trusted the results they were given, but not all were convinced:

*‘*… *and it’s ninety-something per cent accurate, so it would tell us, you know, if there’s any further investigation needed, and it came back okay.’*

(P14, RAPID pathway, aged <65 years)

*‘However, if it’s the case, as I understand, that some thirty or forty per cent of biopsies turn out to be unnecessary it suggests to me that some readings of MRI scans are not correct. That’s the logical conclusion.’*

(P23, traditional pathway, aged ≥65 years)

GPs expressed some uncertainty when assessing how effective prostate MRI is for prostate cancer, potentially stemming from limited knowledge and clinical experience of the test (see intervention coherence above):

*‘… so I don’t know if there are any false negatives or whatever, but, so far, it seems to be working quite well.’*

(GP09, female, RAPID pathway)

Self-efficacy refers to the confidence of the participant that they can complete the activities or behaviours required for the intervention. Patients generally felt they were able to do what was needed to obtain an MRI scan of the prostate:

*‘Oh, fine yes, just lie down and put the ears on. There’s no problems with that.’*

(P03, traditional pathway, aged <65 years)

Access to MRI varies by region, leading to differing views among GPs from different geographical areas as to whether they would be able to order an MRI scan for their patients. This domain also depends on GPs’ level of knowledge and understanding of the test:

*‘Certainly,* […] *I can’t request MRI for anything else, apart from I think we can request them for back … certain back pain and that’s it. Actually, I think there’s just such limited availability for us requesting a … an MRI ever. It’s not something I feel that I have direct access to.’*

(GP05, female, traditional pathway)

*‘We can get most of our patients for most MRIs, generally, within about two to three weeks … ’*

(GP09, female, RAPID pathway)

Some GPs highlighted that prostate MRI was only available to specialists, and was outside their clinical expertise. Exploring the acceptability of prostate MRI on a theoretical basis was more difficult during the interviews with these participants:

*‘No, I mean, it’s … it’s not something that I, sort of … it’s not something that enters my orbit.’*

(GP02, male, traditional pathway)

*‘Well, it’s great, but it’s not available to me. It’s not something I decide on.’*

(GP05, female, traditional pathway)

## Discussion

### Summary

The findings from this qualitative interview study suggest that MRI scanning for possible prostate cancer is acceptable to most patients. The patient interviewees felt generally positive towards having an MRI scan of their prostate. They felt confident they could do what was required of them to undergo an MRI scan, and they had confidence in the ability of MRI to detect prostate cancer. The burden of testing and opportunity cost of attending appointments was significant for patients following the traditional pathway. GPs’ views on the acceptability of MRI scanning for possible prostate cancer were more varied. While some clinicians were supportive, others felt that this diagnostic test was not within their scope of clinical practice, or were worried about patient demand and increased clinical responsibility if it were made available in primary care. Access to MRI is more restricted in Devon, where the traditional pathway is followed, affecting GPs’ self-efficacy.

### Strengths and limitations

This study of patient and GP perceptions of prostate MRI for possible prostate cancer used a published theoretical framework to underpin data analysis. This approach is rare in studies of the acceptability of healthcare interventions to date, as most studies of acceptability are *‘ill-defined, under-theorized, and poorly assessed’*.^15^ Acknowledging the influence of theory and choosing relevant concepts is important in the conduct of healthcare research as it *‘shapes the way practitioners and researchers collect and interpret evidence’*.^21^ A range of views and experiences of the prostate cancer diagnostic pathway and the various tests involved were obtained, via purposively recruiting participants with a range of ages, genders, and geographical locations across two English regions. The researchers adopted a constructivist approach on the assumption that patient and GP participants will experience the pathway in different ways, and this approach appears to be supported by the data collected.

While employing a published theoretical framework to support this analysis could be argued as being a strength, applying it to the GP interviews proved challenging. Sekhon and colleagues^14^ proposed that the framework could be applied prospectively before the intervention had been delivered/received. MRI of the prostate is not currently available for GPs in the UK to order for their patients, so in the analysis of their interviews the subject of acceptability of MRI was prospective in nature. Some GP interviewees were not prepared or able to engage with a discussion about MRI for prostate cancer and were reticent to give their opinion on the acceptability of the test, as it was seen as beyond the scope of their practice. The GPs who did engage sometimes responded to questioning by giving their opinion about how their patients may feel about MRI, rather than from their own perspective. Few data were collected relating to some of the constructs of the framework, particularly for ethicality. There is a wider question about whether Sekhon’s TFA is the most appropriate theory to apply to the analysis of these data. The framework has been developed for the assessment of acceptability of healthcare interventions more broadly, and it could be argued that it is not specific enough for a single test. The TFA is also relatively new and has not yet been widely validated. It may be that it requires some refinement on the basis of more primary data. However, as Sekhon and colleagues highlight in their published work, there are no clearly defined alternatives in existence at this point in time.^15^ This potential limitation was also mitigated by undertaking a broader inductive thematic analysis approach to the entirety of the interviews, recognising that MRI is one test in an extensive diagnostic pathway that does not occur in isolation.

Patients were approached for recruitment into the study after having undergone a prostate MRI as part of an investigation for possible prostate cancer. Six of the patient participants had already been given a diagnosis of prostate cancer by the time the interview was conducted. Most of the remaining patients were awaiting further investigations or had not yet been told the result of the MRI scan. The retrospective assessment of the acceptability of prostate MRI for these patients may have been influenced by the stage of the diagnostic journey they were in at the time of the interview, but there was no clear difference in responses found in the analysis of the interview data.

### Comparison with existing literature

To the authors’ knowledge, this is the first qualitative study to evaluate the acceptability of prostate MRI for detecting prostate cancer. Ullrich and colleagues distributed questionnaires to patients, urologists, and GPs in Düsseldorf, Germany, to assess the acceptance, value, and clinical role of multiparametric MRI (mpMRI) for prostate cancer diagnosis. In total, 328 patients returned their questionnaires, including 251 who had undergone mpMRI, with 223 (68%) considering MRI to be useful, and roughly one-quarter of responders reporting MRI to be constricting, loud, and too expensive.^17^ These responses appear consistent with the experiences of patients in this study; although cost was not raised as a significant concern, which is perhaps unsurprising given that health care is free at the point of care for UK citizens and residents. Ullrich and colleagues’ study did not give a definition for how a test is considered to be ‘useful’. Egbers *et al* assessed the acceptance of MRI-guided biopsy (MRI-GB) in Germany. They performed MRI-GB and TRUS biopsy on 54 patients with suspected prostate cancer, and at least one negative TRUS biopsy. One week later, patients were contacted for a telephone questionnaire that included questions about their preference for MRI-GB or TRUS biopsy, and whether they would undergo MRI-GB again. MRI-GB was the preferred biopsy mode for 35 patients (65%), and 44 patients (81%) said they would undergo MRI-GB again.^16^ Patients in this study reported a preference for MRI over biopsy if given the option, but it was not possible to establish whether the interview participants had had an MRI-GB or TRUS biopsy to compare these biopsy approaches.

In a linked study,^18^ the authors explored the experiences of the whole prostate cancer diagnostic pathway for patients and GPs. ‘Communication’ was a key theme emerging from the patients’ experiences, which in part seemed to influence their understanding of the role of the MRI scan in the diagnostic process and what the results meant. Effective communication with patients regarding diagnostic tests, such as prostate MRI, may influence the level of intervention coherence they possess.

### Implications for research and practice

Direct access to cancer diagnostic testing for GPs for patients with symptoms outside of current guidelines for urgent suspected cancer referral is being implemented by the NHS in England.^7^ If prostate MRI is to be made available in primary care to improve prostate cancer detection and referrals, further research is first needed to clearly define knowledge gaps among GPs regarding prostate cancer diagnosis, and to inform educational interventions and clinical decision support.

The incorporation of pre-biopsy MRI into prostate cancer diagnostic pathways in secondary care has mainly been on the basis of high-quality evidence for improved diagnostic accuracy, and some evidence for cost-effectiveness, relative to TRUS biopsy and in the hospital setting. More recent evidence has also suggested better patient-centred outcomes for MRI compared with TRUS biopsy, including pain, bleeding, and infection.^22^ This study attempted to explore an under-researched area of the implementation of a new diagnostic test into clinical practice, namely acceptability. Sekhon’s TFA provides a clear, evidence-based definition of acceptability and domains within the framework to explore the concept with patients and healthcare staff, even though some of the domains were not easily assessed using the data gathered for this study. Patient and clinician acceptability should be assessed when new diagnostic tests are being tested and implemented in a primary care setting in future.

## Data

Consent was not obtained from participants for sharing of raw data with any parties outside the research team, other than quotations to support findings and conclusions in study reports and peer-reviewed journal articles.
